# Post Discharge mHealth and Teach-Back Communication Effectiveness on Hospital Readmissions: A Systematic Review

**DOI:** 10.3390/ijerph181910442

**Published:** 2021-10-04

**Authors:** Syed Fawad Mashhadi, Aliya Hisam, Siham Sikander, Mommana Ali Rathore, Faisal Rifaq, Shahzad Ali Khan, Assad Hafeez

**Affiliations:** 1 Department of Community Medicine, Army Medical College, National University of Medical Sciences, Rawalpindi 46000, Pakistan; aaleya@yahoo.com (A.H.); mommanaali@gmail.com (M.A.R.); 2 Department of Public Health, Health Services Academy, Opposite National Institute of Health, Islamabad 44000, Pakistan; 3 Global Health Department, Health Services Academy, Opposite National Institute of Health, Islamabad 44000, Pakistan; Siham.Sikander@liverpool.ac.uk; 4 Institute of Population Health, University of Liverpool, Liverpool L69 3BX, UK; 5 Sehat Sahulat Program, Ministry of National Health Services, Regulations and Coordination, Government of Pakistan, Hall 3A, 3rd Floor, Kohsar Block, Pak Secretariat, Islamabad 44000, Pakistan; faisalrifaq@gmail.com; 6 Health Services Academy, Opposite National Institute of Health, Islamabad 44000, Pakistan; Shahzad@hsa.edu.pk (S.A.K.); az10@hsa.edu.pk (A.H.)

**Keywords:** discharge, drug adherence, hospital readmissions, mobile health, teach-back communication, medication adherence

## Abstract

Hospital readmissions pose a threat to the constrained health resources, especially in resource-poor low-and middle-income countries. In such scenarios, appropriate technologies to reduce avoidable readmissions in hospitals require innovative interventions. mHealth and teach-back communication are robust interventions, utilized for the reduction in preventable hospital readmissions. This review was conducted to highlight the effectiveness of mHealth and teach-back communication in hospital readmission reduction with a view to provide the best available evidence on such interventions. Two authors independently searched for appropriate MeSH terms in three databases (PubMed, Wiley, and Google Scholar). After screening the titles and abstracts, shortlisted manuscripts were subjected to quality assessment and analysis. Two authors checked the manuscripts for quality assessment and assigned scores utilizing the QualSyst tool. The average of the scores assigned by the reviewers was calculated to assign a summary quality score (SQS) to each study. Higher scores showed methodological vigor and robustness. Search strategies retrieved a total of 1932 articles after the removal of duplicates. After screening titles and abstracts, 54 articles were shortlisted. The complete reading resulted in the selection of 17 papers published between 2002 and 2019. Most of the studies were interventional and all the studies focused on hospital readmission reduction as the primary or secondary outcome. mHealth and teach-back communication were the two most common interventions that catered for the hospital readmissions. Among mHealth studies (11 out of 17), seven studies showed a significant reduction in hospital readmissions while four did not exhibit any significant reduction. Among the teach-back communication group (6 out of 17), the majority of the studies (5 out of 6) showed a significant reduction in hospital readmissions while one publication did not elicit a significant hospital readmission reduction. mHealth and teach-back communication methods showed positive effects on hospital readmission reduction. These interventions can be utilized in resource-constrained settings, especially low- and middle-income countries, to reduce preventable readmissions.

## 1. Introduction

A patient’s readmission is a financial, social, and psychological burden for the patients and their families [1]. When discharged without adequate education about medication, patients may feel unprepared to care for themselves at home. The Center for Medicare and Medicaid Services defines readmission as “an admission to a subsection hospital within 30 days of discharge from the same or another subsection hospital” [2]. A key factor affecting hospital readmissions is the patient’s inability to comprehend and implement a treatment plan upon discharge. Emphasis must be placed on addressing the issues resulting in readmission for the financial feasibility of public hospitals and the clinical improvement of concerned patients [3]. Demonstrated patient understanding of the discharge plan may decrease the likelihood of readmissions, as high-quality discharge plans are associated with a reduced risk of readmission [4]. Unbiased, transparent, and reliable information improves patient adherence to treatment [5]. One method, called teach-back communication (also known as closing the loop), sees patients trained with small increments of clinical information by hospital staff and then asked to explain in their own words what they have understood [6]. It is a low-cost, evidence-based intervention that can be utilized during hospitalization, discharge, and follow-up and elicits patient–healthcare provider communication [7]. Another method of improving patient compliance is the application of mobile technology in health care. Mobile health (mHealth) is a broad term that refers to any method of patient–provider communication that does not require them to be at the same place. It is also known as telehealth, telemedicine, digital medicine, e-health, or mhealth (short for “mobile health”). Phone calls, video chats, emails, and text messages are used to augment patient communication. With the rapid influx of mobile phones across all socioeconomic strata, mHealth could provide a new and viable model of healthcare [8]. A substantial body of evidence highlights the importance of such cost-effective healthcare delivery approaches for efficient patient management, compliance, self-care, and hospital readmission reduction [9].

Over the past few years, hospital readmissions and their related costs have become a growing concern [10]. According to the Agency for Healthcare Research and Quality, approximately 3.3 million adult, all-cause, 30-day readmissions occurred in the United States in 2011, at a cost of USD 41.3 billion [11]. 

Preventable readmissions create a burden on public hospitals, incurring substantial financial costs at the national exchequer [12]. Conforming to Universal Health Coverage (UHC) and the launch of community health insurance programs in low- and middle-income countries (LMICs), especially South Asia, e.g., Sehat Sahulat Program-Pakistan; Health insurance program—India; National Health Insurance Program—Nepal; Health protection scheme—Bangladesh; Agrahara—Sri Lanka, etc.) have augmented the possibility of increased readmission rates, thus, encumbering the already exhausted healthcare systems [13,14,15,16,17,18]. LMICs significantly lack out-of-hospital care, thereby increasing the likelihood of frequent (re)admissions for even minor ailments [19]. An exhaustive review of existing evidence on readmission-reducing interventions could be instrumental for hospitals in reducing budgetary constraints and improving the quality of post-discharge care [20]. Nexus, this review aims to map the evidence in highlighting the effectiveness of both methods (mHealth and/or teach-back communication) on hospital readmission reduction, summarizes a large amount of information, and informs policymakers and health insurance programs regarding the utilization of low-cost and user-friendly interventions.

## 2. Materials and Methods

This systematic review was conducted following PRISMA (preferred reporting items for systematic reviews and meta-analyses) guidelines [21,22].

### 2.1. Search Strategies

Various search strategies were employed to find as many qualifying studies as possible. The principal author carried out online database searches on PubMed, Wiley Library, and Google Scholar. For each database, search terms were developed, including indexed search terms (e.g., MeSH terms) and keywords in free text. Reference sections of selected articles were also explored to identify relevant articles not retrieved in the online searches (Appendix A).

### 2.2. Study Selection

The articles were required to meet the following inclusion criteria (Table 1): 

(1) Research population included patients having non-communicable diseases.

(2) The study analyzed mHealth/telemedicine and/or teach-back communication as an intervention/exposure.

(3) The outcome of interest was hospital readmissions or frequent hospitalizations reduction.

To identify eligible studies, screening of the titles and abstracts with full text was performed by two independent reviewers (S.F.M. and A.Hi.). In case of disagreement, a third reviewer (S.S) was engaged. Only English language articles published in peer-reviewed journals since 1990 were included. Studies that did not identify an association between teach-back communication and/or mHealth and hospital admission/readmissions were excluded. 

### 2.3. Data Extraction and Management

Using a standardized data collection method, one reviewer (S.F.M.) collected research and patient characteristics, intervention and comparator information, and outcome data from included studies. Three authors (S.A.K., M.A.R., and F.R.) double-checked the work for accuracy, and any discrepancies were settled by consensus. Duplicate publications of the same study were screened for additional data and, if necessary, authors were contacted. A table for data extraction was developed through discussion between the authors and was designed to capture extract author(s) name, publication year, country, sample size, study design, intervention, condition, and key findings from the selected studies. The principal investigator extracted data on an excel sheet which was then verified by the second evaluator. 

### 2.4. Assessment of Quality of Studies

Quality assessment of studies was carried out using the Quality Assessment Criteria for Evaluating Primary Research Papers from a Variety of Fields (QualSyst tool for the quantitative studies) by two authors (S.F.M. and A.Hi) independently [23,24] QualSyst developed by Kmet et al. [25] ensures that finally selected researches that form the basis of the review, conform to a minimum quality standard. The quantitative component of the Qualsyst tool incorporating a scoring system using 14 items has been utilized in conducting this review. Published papers were assigned a score of 2, 1, or 0 for each question depending upon whether they satisfy (Yes), partially satisfy (Partial), or do not satisfy (No) the specified question. In the quantitative tool, ‘not applicable (N/A)’ can also be selected for some questions. The obtained total score was divided by 18 to 28 (total possible score) depending upon the “N/A” options selected. The obtained score was then multiplied by 100 to provide a summary quality score (SQS), expressed as a percentage. The average of the two reviewers’ independent scores was calculated to find out the average SQS (first adding the independent scores from each author then dividing the sum by 2 to get the mean). A higher degree of methodological vigor is depicted by a higher percentage. The QualSyst tool for quantitative studies is attached as Appendix B. 

## 3. Results

### 3.1. Search Strategy and Study Selection

**Figure 1:**
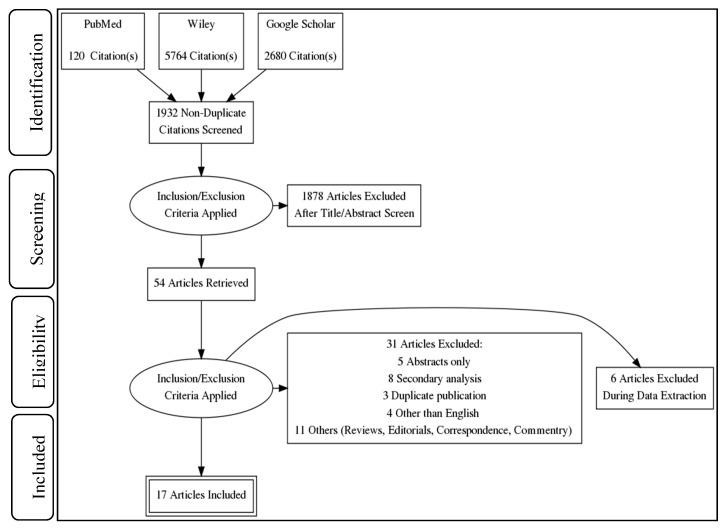
PRISMA flow diagram. The implementation of inclusion and exclusion criteria in a step-by-step manner determined the total number of studies in the systematic review.

Our search retrieved 8564 articles from three search engines, i.e., PubMed, Wiley, and Google Scholar. After removing duplicates (6632 articles), a total of 1932 articles were selected for further processing. Three authors (S.F.M., F.R., and M.A.R.) independently screened the titles and abstracts of these manuscripts and found 54 articles. After applying eligibility criteria to these 54 articles, 37 were found to be ineligible. At the time of data extraction and further scrutiny of these articles, a total of 17 studies were selected for this review (Figure 1).

### 3.2. Studies Characteristics

The total number of participants in these 17 included studies was 5713. The studies were published between 2002 and 2020. Of 17 publications, 8 were carried out in the USA, 2 each in Australia, and Denmark and one each in the rest of the counties namely the United Kingdom (UK), Iran, Spain, Belgium, and China. We found different interventions intended to reduce hospital (re)admissions. All the studies specified hospital admissions/readmissions reduction as the primary outcome. Funding sources were not documented in all publications.

Of all the included studies (*n* = 17), 52.9% (*n* = 9) were randomized controlled trials (RCT), cohort studies (CS) (17.6%, *n* = 3), quasi experimental studies (17.6%, *n* = 3) while other studies were 11.8% (*n* = 2).

mHealth was utilized in 11 studies out of which, 6 studies were RCTs, 4 studies were quasi-experimental while one was a CS. Teach-back communication as an intervention/exposure strategy was utilized in 6 studies out of which 3 were RCTs, 2 were CS and 1 study was a cross-sectional study (miscellaneous). Among the studies utilizing mHealth (*n* = 11), 7 studies [26,27,28,29,30,31,32] showed significant reduction (*p* < 0.05) in hospital readmissions while four studies [33,34,35,36] did not show any significant reduction (*p* > 0.05) in readmissions. Among the teach-back communication group (*n* = 6), the majority of the studies (*n* = 5) [37,38,39,40,41] showed a significant reduction in hospital readmissions while one publication [42] did not show any significant hospital readmission reduction. Of 17 publications, 41.2% (*n* = 7) were targeted towards heart failure (HF), 23.5% (*n* = 4) encompassed chronic obstructive pulmonary disease (COPD), 23.5% (*n* = 4) multiple chronic conditions, while 11.8% (*n* = 2) publications utilized other conditions (hip and knee arthroplasties and type 2 diabetes mellitus). Detailed characteristics of the included studies are shown in (Table 2).

### 3.3. Studies Quality Assessment

**Figure 2:**
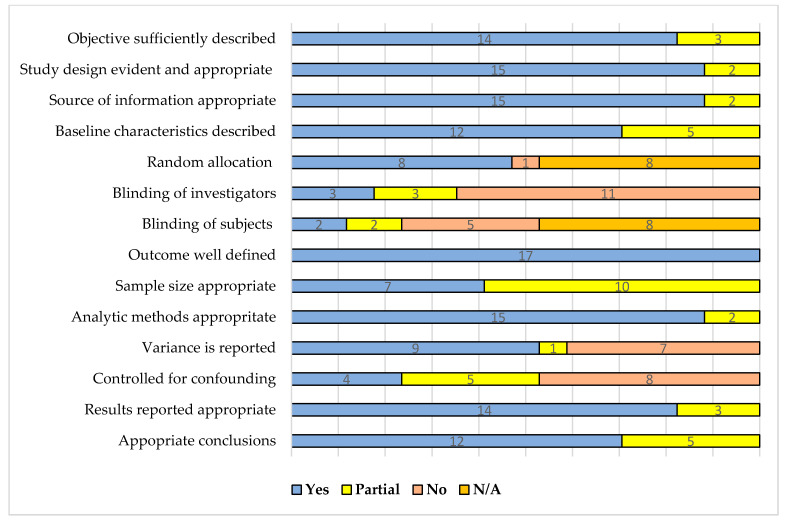
Summarized quality assessment of the included studies (*n* = 17) using QualSyst tool.

The majority of the studies had high-quality scores as assessed by the QualSyst tool independently by two reviewers (S.F.M. and A.Hi.). Only three parameters (controlled for confounding, variance report, and blinding) were not accurately described in a few publications. Figure 2 shows the summary of the quality assessment utilizing the QualSyst tool for quantitative studies. The detailed scoring matrix is attached as Appendix C.

The average SQS of all the included studies was 81% (range = 57–95%). The average SQS of the studies utilizing mHealth was 78% (range = 57–95%, *n* = 11) while the average SQS of teach-back communication publications was 86% (range = 82–91%, *n* = 6). The average SQS of mHealth studies (*n* = 7) showing a significant reduction in the outcome parameter (reduction in hospital readmissions) was 76% (range = 57–95%) while the average SQS of mHealth studies not showing a significant reduction in the hospital readmissions (*n* = 4) was 81% (range = 71–93%). The average SQS of teach-back communication studies showing a significant reduction in the outcome parameter (*n* = 5) was 86% (range = 82–91%) while studies showing no significant reduction in the outcome parameters (*n* = 1) had an SQS of 82%. The detailed average SQS statistics are shown in Appendix D.

## 4. Discussion

Hospital readmissions pose a voluminous challenge to limited health services, especially in resource-constrained LMICs. In such circumstances, the use of appropriate technology to minimize readmissions in hospitals necessitates more creative and efficient intervention strategies. We identified the prevalence of two different components, mHealth and teach-back communication and both of these exhibited effectiveness in hospital readmissions reduction among a total of eight countries over 17 years. Although most of the studies were conducted in high-income countries, the appropriate use of mHealth and teach-back communication in LMICs may prove beneficial. mHealth was analyzed in 64.7% (11 out of 17) while teach-back communication was observed in 33.3% (6 out of 17) of the included studies. Most studies were conducted in the USA. The majority were RCTs but a variety of other study designs were also employed. The use of mHealth was significantly associated with a decrease in hospital readmissions, whereas the readmission rate also decreased by almost half when using teach-back communication. This review highlights that mHealth and teach-back communication are two effective interventions for reducing avoidable hospital readmissions.

This review has identified qualitative evidence of mHealth effectiveness on hospital readmission reduction. Teach-back should be paired with other readmissions reducing programs of a hospital as it can affect 30-day readmission outcomes [43]. It is low cost, requires little extra staff time, and can have a favorable impact on patients with chronic diseases. Only one of the six studies had a patient-needs assessment of the intervention component. Additionally, only two studies included phone calls. The distribution and quantity of these studies especially in low- and middle-income countries suggest that very little research has been carried out on teach-back communication. The role of health literacy should be considered in guaranteeing high-quality patient-centered care.

In 2009, the Centers for Medicare and Medicaid Services started reporting risk-standardized 30-day readmission rates as a measure of hospital quality. In the fiscal year 2012, these programs implemented a financial penalty on the hospitals with a high incidence of readmissions for pneumonia, congestive heart failure, or acute myocardial infarction patients [44].

Several multi-component approaches have effectively lowered readmission rates for patients discharged to home (e.g., patient needs assessment, prescription reconciliation, patient education, scheduling timely outpatient appointments, and offering telephone follow-up) [45,46]. The evidence from the included studies indicates that mHealth and teach-back have a clear and beneficial impact. These results are in line with another systematic review [47,48] in which mHealth provided extensive and context-sensitive support for hospital readmission reduction among heart failure patients.

Astetxe conducted a systematic review in which he has proposed predictive models for hospital readmission risk. In his review, a total of 265 publications were reviewed and 77 studies were selected. The predictive models facilitated the identification of potentially high-risk individuals [49], while in this review, the objective was to evaluate the effectiveness of interventions on hospital readmission among patients with chronic non-communicable diseases. Further studies can be conducted in line with Astetxe to predict high-risk readmission.

The effect of interventions on readmission rates is related to the number of components implemented; single-component interventions are unlikely to reduce readmissions significantly. For patients discharged to post-acute care facilities, multicomponent interventions have reduced readmissions through enhanced communication, medication safety, advanced care planning, and enhanced training to manage medical conditions that commonly precipitate readmission [50]. Additionally, this study enhances the body of evidence related to the importance of mHealth and teach-back in affecting readmission reduction. 

In a review, the patients who belonged to congestive heart failure (CHF), renal failure, urinary tract infection (UTI), pneumonia, and COPD are examples of typical initial (“index”) diagnoses for hospitalizations and subsequent readmissions [51]. While in another review, patients with cardiac failure (26.7%), psychoses (24.6%), recent vascular surgery (23.9%), chronic obstructive pulmonary disease (22.6%), and pneumonia had the highest 30-day readmission rates (20.1%) [50]. The findings of a feasibility study suggested that mHealth features may be useful in predicting unplanned readmissions [52]. Another research focused on the possible benefits of mHealth interventions in LMICs [53].

Patients also have difficulty comprehending or remembering information presented by their healthcare providers. Recognized as “say back” or “show me”, teach-back communication operates better when healthcare providers hold themselves responsible rather than the patient for the latter’s lack of comprehension [54]. Teach-back has been shown to improve patients’ skills and self-care ability, but there is no guidance for healthcare organizations trying to adopt it [55]. Teach-back is the most commonly used approach as part of a structured yet simple strategy, with this method of “teach-back enhanced education” being found to be useful in a wide range of contexts, populations, and outcomes. The locations included hospitals, outpatient clinics, the emergency department, and community health centers. Many health interventions are tailored for a specific environment and are rarely used outside of that environment. The results of this study reveal that teach-back enhanced education is widely used in a number of settings, including the emergency room [56].

Teach-back has been found to help individuals with chronic diseases in improving their knowledge, skills, and self-care capacities, but there is no guidance for healthcare organizations trying to adopt it. Although teach-back is the most commonly used approach as part of a structured yet simple strategy, this method of “teach-back enhanced education” has been found to be useful in a wide range of contexts, populations, and outcomes [54,55]. The results of this review also provide evidence in favor of teach-back, showing that teach-back enhanced education impacts hospital readmission reduction in a number of settings, different study designs, and diverse populations. 

The emphasis of the analysis was on research that analyzed the effect of teach-back and mHealth on readmission reduction at the hospital level using administrative data. This could assist health policymakers in developing reliable methodologies and management strategies for patients at high risk of readmission at a regional level. Different risk factors have been evaluated in previous selected clinical trials, but they had the drawback of a small number of patients and a shorter follow-up duration. Further studies focusing on cause-specific readmission rates can allow a broader sample of patients with a broader clinical picture of readmission reduction. 

The Hospital Readmissions Reduction Program is a Medicare value-based purchasing program that enables hospitals to handle correspondence and care coordination in order to better engage patients and caregivers in discharge plans, reducing unnecessary readmissions. The program contributes to the national goal of enhancing health care for patients by tying payment to the quality of hospital services [57]. In 2009, the Centers for Medicare and Medicaid Services started reporting risk-standardized 30-day readmission rates as a measure of hospital quality. They implemented a payment scheme in the fiscal year 2012 that penalizes hospitals with a high incidence of readmissions for pneumonia, congestive heart failure, or acute myocardial infarction (AMI) patients [58]. This strategy can be adopted in developing countries such as Pakistan to decrease the hospital readmissions in private and public sector hospitals. 

Mobile phones were primarily used for the sending and receiving of short message services (SMS). These studies also reported positive evaluations of using mobile phone-based interventions. It was found that 70% of participants viewed the SMS intervention as positive and only 10% held negative views [59]. These findings are important and demonstrate that even simple mobile devices can be used for interventions using functions such as SMS alerts, voice calling, or alarms. This is relevant for low resource settings, where large populations may have access to mobile devices with basic functionality [54].

An increasing number of top hospitals are implementing mHealth—the use of mobile technology devices and smartphones for healthcare—to link patients and physicians, improve care management, and minimize avoidable, expensive hospital readmissions. mHealth improves chronic disease management outcomes, treatment planning, and data collection for population health management by directly addressing communication gaps in the healthcare delivery system [60].

Four mHealth studies [33,34,35,36] in this review have shown either no or non-significant effects on the hospital readmission reduction. Their quality assessment through the QualSyst tool was within acceptable limits, however, there were wide variations in their methodology and reporting, though the sample size was also large, ranging from 205 to 1437 participants. The results of these trials are contradictory to this systematic review as the majority of the selected studies showed a positively significant reduction in hospital readmissions, while one [42] teach-back study concluded no significant reduction. The sample size of teach-back was almost similar to mHealth ranging from 88 to 1033 participants. These trial results are in contradiction to this systematic review as the majority of the selected studies (seven mHealth and five teach-back) showed a positive significant reduction in hospital readmissions. 

The review has its own limitations as the search was restricted to published research, there was a risk of publication bias in this study. Furthermore, a meta-analysis was not feasible due to the heterogeneity of readmission rates such as a reduction in the number of days, 30 days readmission reduction, 180 days readmission reduction, etc. In addition, heterogeneity was also observed in the research designs. This might result in an overestimation or underestimation of the effects of the interventions.

Understanding the relationship between implementation and health outcomes would be beneficial; however, due to the lack of detail about implementation and the heterogeneity of implementation strategies in the included studies, this was not possible. 

We searched for currently available health care strategies to reduce readmissions among patients with chronic diseases, without selecting quality studies. Our advantage was the wide range of included literature, from several sources, a careful review process and quality assessment using a validated QualSyst tool by three reviewers. To the best of our knowledge, we are the first in our country to undertake a systematic review and evidence mapping on the subject. We were limited by extracting only 17 studies meeting the eligibility criteria, as scant research on this subject is available. Not all published articles or gray literature may have been included as searching from other sources may have included additional publications. 

### 4.1. Health System Strategy and Policy Implications

This research has a wide range of health system and policy implications. To begin with, healthcare managers need to ensure that they are systematically incorporating data against the cause of readmission in order to enhance care coordination, eliminate avoidable readmission rates, and maximize the usage and access to critical patient information in the future. This study indicates that regional health information education has very little infiltration into health systems, at least in terms of readmission reduction measures. This could be a significant opportunity to strengthen utilization and long-term feasibility for mHealth or teach-back communication interventions. 

### 4.2. Future Research Implications

This systematic review provides evidence-based interventions to reduce readmissions, especially in constrained public and private sectors health service delivery systems. Policymakers and healthcare managers, at all tiers, can utilize these low-cost and effective interventions to reduce the burden of preventable readmissions by connecting with the patient through the health system. Long-term follow-up studies centered solely on risk factors linked to a higher readmission rate can be planned. A crude cross-sectional survey of the entire population to assess common causes of readmission can be undertaken. The high risk of readmission such as HF, type II diabetes, hypertension, etc., can be specifically targeted for mHealth. Emerging advances in technology through automated reminders and social media platforms should be considered for additional evaluation. Larger RCTs, in the context of LMICs, are required to determine the effectiveness of low-cost strategies such as teach-back and /or mHealth in reducing readmissions, frequent hospitalizations, and improvement in patient outcomes.

## 5. Conclusions

Although mHealth has evolved over a period, its efficacy and bearing on the healthcare service delivery in most countries around the globe are limited. At the same time, teach-back communication is a well-defined structured intervention that not only enhances patient health literacy but also augments patient–provider interaction. SMS reminders, telephone calls, and teach-back communication have demonstrated positive effects in multiple chronic diseases such as improving chronic pulmonary disease symptoms and heart failure conditions, by reducing preventable readmissions, and enhancing medications adherence. The methodological rigor of the studies included in this systematic review is generally of high quality. For some conditions, the interventions have not demonstrated efficacy, which could be due to variations in the study designs, different sample sizes, and various disease states.

## Figures and Tables

**Table 1:** Population, intervention, control, outcome, and time period—PICO(T).

Population	
Age	>20 years of age
Gender	Both genders
Disease status	Patients with non-communicable diseases (NCDs)
Location	Any country and region
Intervention/Exposure	
mHealth/telemedicine/telehealth	Mobile health communication through telephone and/or short text messages and/or Delivery of health services via remote telecommunications
Teach-back communication	Patient education and (or) information about discharge instructions allowing them to restate the instructions in their own words.
Setting	Outpatient department, admitted cases, or both
Control	
Standard care	Patients with routine/usual care
Outcome	
Primary outcome	Hospital readmissions or frequent hospitalizations reduction
Period of observation	30–180 days after index discharge
Study Design	
Study design included	Quantitative study design
Time Period	
Searched till	June 2020
Exclusion Criteria	Duplicate publication
	Articles not specifying hospital readmission reduction
	Where full-text articles could not be recovered.
	Studies that were neither available in English nor could be translated
	Studies that have utilized secondary data analysis
	Qualitative studies, opinion pieces, theoretical papers, non-peer-reviewed manuscripts, abstracts, reviews, editorials, commentaries, correspondence.

**Table 2:** Characteristics of included studies (*n* = 17).

Author, Publication Year	Country	Design	Condition	Sample Size (*n*)	Intervention	Key Findings
Celler B et al. 2017 [26]	Australia	BACI	Multiple chronic conditions	237	mHealth	Intervention group showed a 53.2% reduction in the rate of predicted unscheduled readmission to hospital (*p* = 0.02) and a reduction in mortality between 41.3% and 44.5% as compared to the controls. Statistical tests: Chi-square test, Fisher exact test for categorical variables, the two-sample *t*-test for continuous variables, Wilcoxon rank-sum test for skewed variables. Quality score: 95%
Dastoon M et al. 2016 [37]	Iran	RCT	HF	100	Teach-back communication	Greater time spent in teach-back communication significantly reduced hospital readmissions by 56.2% in the intervention group (44 vs. 21, *p* = 0.04). Statistical tests: Man–Whitney U and Chi-square tests Quality score: 82%
De Walt DA et al. 2006 [38]	USA	RCT	HF	123	Teach-back communication	Intervention group had a decreased rate of hospitalization [adjusted incidence rate ratios (IRR)] = 0.53; CI 0.32, 0.89). Statistical technique: Multivariate regression analysis Quality score: 82%
Dinesen B et al. 2012 [27]	Denmark	RCT	COPD	111	mHealth	Intervention group demonstrated a significantly reduced (*p* = *0*.04) requirement of hospitalization and 30-day readmissions. Statistical tests: Kaplan–Meier survival analysis, log rank test Quality score: 79%
Estaban C et al. 2016 [28]	Spain	NR-OS	COPD	197	mHealth	Intervention group had significantly lower rates of 30 days readmission (OR = 0.46, 95% CI = 0.29–0.74; *p* < 0.001). Statistical tests: Chi-square test for qualitative variables and a two-sampled Wilcoxon test for continuous variables. Quality score: 77%
Frederix I et al. 2018 [32]	Belgium	Multicenter prospective RCT	HF	142	mHealth	The number of days lost due to readmissions was significantly lower in the intervention group (*p* = 0.04). Statistical tests: Independent *t*-tests (parametric) or Mann–Whitney U tests (nonparametric) for continuous variables and Chi-square test for categorical variables, Cox regression model for hazards ratio Quality score: 64%
Greenup EP et al. 2017 [33]	Australia	Clinical trial (non-randomized)	Multiple chronic conditions	345	mHealth	No significant difference in rates of readmission in intervention group. Statistical tests: Chi-square test, binary logistic regression model. Quality score: 77%
Howie-Esquivel J et al. 2015 [39]	USA	Cross-sectional	HF	1033	Teach-back communication	Usual care group was 1.5 times more likely to be hospitalized (95% CI: 1.2–1.9; *p* = 0.001) compared to intervention group. Statistical technique: Multiple logistic regression. Quality score: 91%
Krumholz HM et al. 2002 [40]	USA	RCT	HF	88	Teach-back communication	After adjusting for clinical and demographic characteristics, the intervention group had a significantly lower risk of readmission as compared with the control group (Hazard ratio = 0.56, 95% CI: 0.32, 0.96; *p* = 0.03) Statistical tests: Mantel–Haenszel chi-square, Cox proportional hazards model. Quality score: 89%
Ong MK 2016 [34]	USA	RCT	HF	1437	mHealth	Telephone calls and TM did not reduce 180-day readmissions. Statistical technique: Multivariate logistic regression. Quality score: 93%
Pinnock H et al. 2013 [35]	UK	RCT	COPD	256	mHealth	TM was not effective in postponing hospital readmission for patients with ECOPD. Statistical technique: Kaplan–Meier survival analysis, using Cox proportional hazards model. Quality Score: 82%
Rosen OZ et al. 2017 [41]	USA	CS	Multiple chronic conditions	385	Teach-back communication	Patients with combined low and intermediate adherence had readmission rates of 2% compared to 9.3% for patients with high adherence (*p* = 0.05) Statistical tests: Wilcoxon rank-sum test and Chi-square test Quality score: 91%
Rosnar BI et al. 2018 [29]	USA	Multicenter CS	Hip and knee arthroplasties	558	mHealth	A statistically significant reduction in readmission rate in the mHealth arm (3.4%; 95% CI, 0.1–6.7%) vs the control (12.2%; 95% CI, 6.4–18.0%) (*p* = 0.01). Statistical tests: Fisher’s exact test and *t*-test Quality score: 86%
Sorknaes AD et al. 2011 [30]	Denmark	Clinical trial (non-randomized)	COPD	100	mHealth	In intervention group TM consultation resulted in 12% readmissions vs 22% in control group, days of readmission were reduced by about 20 days. Statistical Tests: Kaplan–Meier survival analysis and multivariate Cox regression analysis Quality score: 57%
Takahashi PY et al. 2012 [36]	USA	RCT	Multiple chronic conditions	205	mHealth	No statistical difference was noted in hospitalizations and ER visits between the TM group (63.7%) and the group receiving usual care (57.3%) (*p* = 0.345) Statistical tests: Wilcoxon rank sum test, two-sample *t*-test and Chi- squared test Quality score: 71%
Wang Y et al. 2019 [31]	China	RCT	Type 2 diabetes	120	mHealth	Intervention significantly (*p* < 0.05) reduced hospitalization in the intervention group. Statistical tests: Chi-squared tests for categorical variables and independent sample *t*-tests for continuous variables Quality score: 75%
White M et al. 2013 [42]	USA	CS	HF	276	Teach-back communication	No statistical significance (*p* = *0*.775 and 0.609) was observed either in patients who answered teach-back questions correctly or in the reduction of 30-day hospital readmission rates. Statistical Tests: Chi-squared test for categorical data; Fisher exact test for dichotomous data, and Student *t-*test to compare quantitative data Quality score: 82%

BACI: Before and after control intervention, CI: Confidence Interval, COPD: Chronic obstructive pulmonary disease, CS: cohort study, ECOPD: Exacerbation of chronic obstructive pulmonary disease, HF: heart failure, RCT: randomized controlled trial, NR-OS: non-randomized observational study, TM: Telemedicine, OR: Odds ratio.

## Data Availability

The data presented in this study are available on request from the corresponding author.

## Appendix A

This appendix shows the detailed online search strategy used for online search of articles.

**Date: May 2021**		
**Keywords/Mesh Terms**	**Query**	**Items Found**
**Pubmed**		
1.	(mHealth) OR (mobile health) OR (Telehealth) OR (Telecommunication)	153,267
2.	(Teach-back ) OR (Teach-back experience) OR (Teach-back communication) OR (Closing the loop) OR (Discharge counselling)	320
3.	(Hospital readmission reduction) OR (Hospital readmissions reduction) OR (Readmission reduction) OR (Frequent admissions reduction) OR (frequently admitted patients reduction)	3851
4.	(((Teach-back ) OR (Teach-back experience) OR (Teach-back communication) OR (Closing the loop) OR (Discharge counselling)) OR ((mhealth) OR (mobile health) OR (Telehealth) OR (Telecommunication))) AND (((Hospital readmission reduction) OR (Hospital readmissions reduction) OR (Readmission reduction) OR (Frequent admissions reduction) OR (frequently admitted patients reduction)))	120
**Wiley**		
1.	(mhealth) OR (mobile health) OR (Telehealth) OR (Telecommunication)	138
2.	(Teach-back ) OR (Teach-back experience) OR (Teach-back communication) OR (Closing the loop) OR (Discharge counselling)	332,905
3.	(Hospital readmission reduction) OR (Hospital readmissions reduction) OR (Readmission reduction) OR (Frequent admissions reduction) OR (frequently admitted patients reduction)	21,751
4.	(((Teach-back ) OR (Teach-back experience) OR (Teach-back communication) OR (Closing the loop) OR (Discharge counselling)) OR ((mhealth) OR (mobile health) OR (Telehealth) OR (Telecommunication))) AND (((Hospital readmission reduction) OR (Hospital readmissions reduction) OR (Readmission reduction) OR (Frequent admissions reduction) OR (frequently admitted patients reduction)))	5764
**Google scholar**		
1.	(mhealth) OR (mobile health) OR (Telehealth) OR (Telecommunication)	3,940,000
2.	(Teach-back ) OR (Teach-back experience) OR (Teach-back communication) OR (Teach-back) OR (Teach-back communication)	8960
3.	(Hospital readmission reduction) OR (Hospital readmissions reduction) OR (Readmission reduction) OR ( readmissions reduction)	66,900
4.	(((Teach-back ) OR (Teach-back experience) OR (Teach-back communication) OR (Closing the loop) OR (Discharge counselling)) OR ((mhealth) OR (mobile health) OR (Telehealth) OR (Telecommunication))) AND (((Hospital readmission reduction) OR (Hospital readmissions reduction) OR (Readmission reduction) OR (Frequent admissions reduction) OR (frequently admitted patients reduction)))	2680
**Grand Total**		**8564**

## Appendix B

QualSyst tool for quantitative studies.

**Serial**	**Questions for Quantitative Scoring**	**Yes** **(2)**	**Partial** **(1)**	**No** **(0)**	**N/A**
1.	Question/objective sufficiently described?				
2.	Study design evident and appropriate?				
3.	Method of subject/comparison group selection or source of information /input variables described and appropriate?				
4.	Subject (and comparison group, if applicable) characteristics sufficiently described?				
5.	If interventional and random allocation was possible, was it described?				
6.	If interventional and blinding of investigators was possible, was it reported?				
7.	If interventional and blinding of subjects was possible, was it reported?				
8.	Outcome and (if applicable) exposure measure(s) well defined and robust to measurement/misclassification bias? Means of assessment reported?				
9.	Sample size appropriate?				
10.	Analytical methods described/justified and appropriate?				
11.	Some estimate of variance is reported for the main results?				
12.	Controlled for confounding?				
13.	Results reported in sufficient detail?				
14.	Conclusions supported by the results?				

## Appendix C

Detailed matrix of quality assessment of included studies (*n* = 17) using QualSyst tool for quantitative studies.

	**Total**				**Study 1**	**Study 2**	**Study 3**	**Study 4**	**Study 5**	**Study 6**	**Study 7**	**Study 8**	**Study 9**	**Study 10**	**Study 11**	**Study 12**	**Study 13**	**Study 14**	**Study 15**	**Study 16**	**Study 17**
**Criteria**	**Yes**	**Partial**	**No**	**NA**	**Celler B et al**	**Dastoon M et al**	**De Walt et al**	**Dinesen B et al**	**Estaban C et al**	**Frederix et al**	**Greenup EP et al**	**Howie-Esquivel J et al**	**Krumholz HM et al**	**Ong MK**	**Pinnock H et al**	**Rosen OZ et al**	**Rosnar BI et al**	**Sorknaes ED et al**	**Takahashi PY et al **	**Wang Y et al**	**White M et al**
Objective sufficiently described	14	3	0	0	2	2	1	1	1	2	2	2	2	2	2	2	2	2	2	2	2
Study design evident and appropriate	15	2	0	0	2	2	2	2	1	2	1	2	2	2	2	2	2	2	2	2	2
Source of information appropriate	15	2	0	0	2	2	2	2	2	1	1	2	2	2	2	2	2	2	2	2	2
Baseline characteristics described	12	5	0	0	2	2	2	2	2	1	1	2	2	2	2	2	2	1	1	1	2
Random allocation	8		1	8	NA	2	2	2	NA	2	NA	NA	2	2	2	NA	NA	0	2	2	NA
Blinding of investigators	3	3	12	0	NA	2	2	2	NA	2	NA	NA	2	2	2	NA	NA	0	2	1	NA
Blinding of subjects	2	2	5	8	NA	1	1	2	NA	2	NA	NA	0	0	0	NA	NA	0	0	2	NA
Outcome well defined	17	0	0	0	2	2	2	2	2	2	2	2	2	2	2	2	2	2	2	2	2
Sample size appropriate	7	10	0	0	1	1	2	1	2	1	1	1	1	2	2	2	2	1	1	1	2
Analytic methods appropriate	15	2	0	0	2	2	2	1	2	1	2	2	2	2	2	2	2	2	2	2	2
Variance is reported	9	1	7	0	2	2	2	2	2	0	2	2	2	2	1	0	0	0	0	0	0
Controlled for confounding	4	5	8	0	2	0	1	0	0	0	1	1	2	2	1	2	1	0	0	0	0
Results reported appropriate	14	3	0	0	2	2	1	1	2	1	2	2	2	2	2	2	2	2	2	2	2
Conclusions appropriate	12	5			2	1	1	2	1	1	2	2	2	2	1	2	2	2	2	2	2
**Summary Score**	**-**	**-**	**-**	**-**	**95**	**82**	**82**	**79**	**77**	**64**	**77**	**91**	**89**	**93**	**82**	**91**	**86**	**57**	**71**	**75**	**82**
**Total score/Numerator**	147	43	33	16	21	23	23	22	17	18	17	20	25	26	23	20	19	16	20	21	18
**Denominator**	28	14			22	28	28	28	22	28	22	22	28	28	28	22	22	28	28	28	22

## Appendix D

Summary Quality Score Statistics (SQS) for included studies.

**Studies Included in the Systematic Review**	**Total Participants**	**SQS (%)**	
		**Average**	**Range**
All (*n* = 17)	5713	81	57–95
- With significant outcome (*n* = 12)	3194	81	57–95
- Without significant outcome (*n* = 5)	2519	81	71–93
- RCTs (*n* = 9)	2582	80	64–93
- With significant outcome (*n* = 6)	684	79	64–89
- Without significant outcome (*n* = 3)	1898	82	71–93
- Others (*n* = 8)	3131	82	57–95
- With significant outcome (*n* = 6)	2510	83	57–95
- Without significant outcome (*n* = 2)	621	80	77–82
mHealth (*n* = 11)	3708	78	57–95
- With significant outcome (*n* = 7)	1465	76	57–95
- Without significant outcome (*n* = 4)	2243	81	71–93
- RCTs (*n* = 6)	2271	77	64–93
- With significant outcome (*n* = 3)	373	71	64–79
- Without significant outcome (*n* = 3)	1898	82	71–93
- Others (*n* = 5)	1437	78	57–95
- With significant outcome (*n* = 4)	1092	79	77–95
- Without significant outcome (*n* = 1)	906	77	-
Teach—Back Communication (*n* = 6)	2005	86	82–91
- With significant outcome (*n* = 5)	1729	87	82–91
- Without significant outcome (*n* = 1)	276	82	-
- RCTs (*n* = 3) *	311	84	82–89
- Others (*n* = 3)	1694	88	82–91
- With significant outcome (*n* = 2)	1418	91	-
- Without significant outcome (*n* = 1)	276	82	-

* All the RCTs in teach-back communication group had statistically significant outcome.
